# The expression and functional activities of smooth muscle myosin and non‐muscle myosin isoforms in rat prostate

**DOI:** 10.1111/jcmm.13345

**Published:** 2017-10-09

**Authors:** Ping Chen, Jing Yin, Yu‐ming Guo, He Xiao, Xing‐huan Wang, Michael E. DiSanto, Xin‐hua Zhang

**Affiliations:** ^1^ Department of Urology Zhongnan Hospital of Wuhan University Wuhan China; ^2^ Department of Rehabilitation Zhongnan Hospital of Wuhan University Wuhan China; ^3^ Department of Surgery and Biomedical Sciences of Cooper Medical School of Rowan University Camden NJ USA

**Keywords:** prostate, smooth muscle, myosin, non‐muscle myosin, isoforms

## Abstract

Benign prostatic hyperplasia (BPH) is mainly caused by increased prostatic smooth muscle (SM) tone and volume. SM myosin (SMM) and non‐muscle myosin (NMM) play important roles in mediating SM tone and cell proliferation, but these molecules have been less studied in the prostate. Rat prostate and cultured primary human prostate SM and epithelial cells were utilized. *In vitro* organ bath studies were performed to explore contractility of rat prostate. SMM isoforms, including SM myosin heavy chain (MHC) isoforms (SM1/2 and SM‐A/B) and myosin light chain 17 isoforms (LC_17a/b_), and isoform ratios were determined *via* competitive RT‐PCR. SM MHC and NM MHC isoforms (NMMHC‐A, NMMHC‐B and NMMHC‐C) were further analysed *via* Western blotting and immunofluorescence microscopy. Prostatic SM generated significant force induced by phenylephrine with an intermediate tonicity between phasic bladder and tonic aorta type contractility. Correlating with this kind of intermediate tonicity, rat prostate mainly expressed LC_17a_ and SM1 but with relatively equal expression of SM‐A/SM‐B at the mRNA level. Meanwhile, isoforms of NMMHC‐A, B, C were also abundantly present in rat prostate with SMM present only in the stroma, while NMMHC‐A, B, C were present both in the stroma and endothelial. Additionally, the SMM selective inhibitor blebbistatin could potently relax phenylephrine pre‐contracted prostate SM. In conclusion, our novel data demonstrated the expression and functional activities of SMM and NMM isoforms in the rat prostate. It is suggested that the isoforms of SMM and NMM could play important roles in BPH development and bladder outlet obstruction.

## Introduction

Benign prostatic hyperplasia (BPH) is the most common pathologic process causing lower urinary tract symptoms (LUTS) and erectile dysfunction (ED) in ageing men with a histologic prevalence of approximately 50% for men in their 60s, and 90% for men in their 80s 1. Although the aetiology of BPH is not well understood, androgens and ageing are necessary for the development of BPH 2 containing two physiological components: static (increased prostate size) 3 and dynamic (increased prostatic smooth muscle (SM) tone) 4. The current standard pharmacotherapy for bothersome BPH/LUTS is α1‐adrenergic antagonists (α‐blockers) and antiandrogenic 5α‐reductase inhibitors, targeting the dynamic and static components, respectively 2, 5. However, 5α‐reductase inhibitors are only indicated for patients with prostate volumes over 40 ml. Thus, dynamic components may play a more important role in BPH.

**Figure 1:**
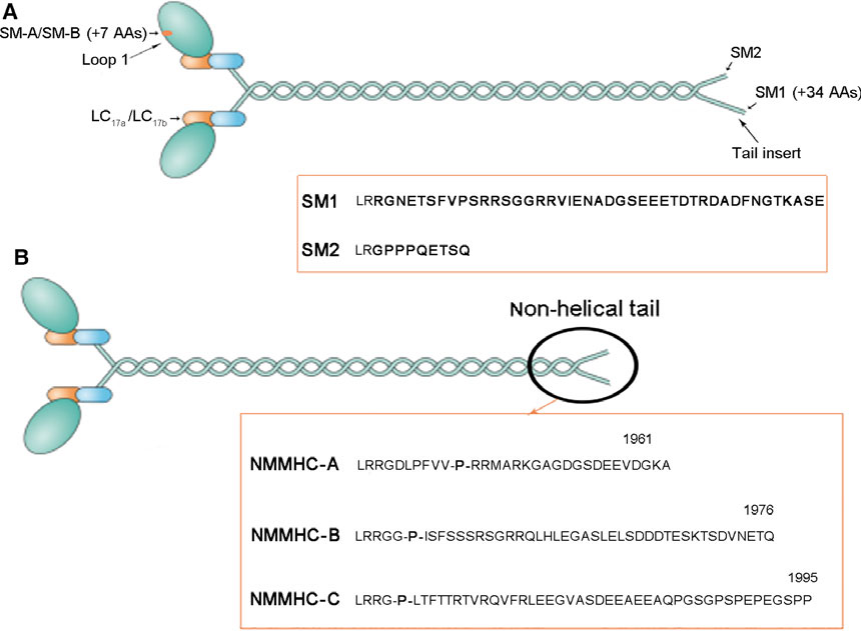
The schematic figure of isoform‐specific differences between the SMM, NMM and LC_17_ isoforms. (A) The isoform‐specific differences of SMM. The SM MHC subunits derive from two alternative splices, one is a length difference of 34 amino acids (AAs) (a sequence difference of 43 AAs) at the non‐helical COOH terminus of the MHC (tail insert; SM1 is the longer tail and SM2 is the shorter tail) and the other one is the addition of 7 AAs at loop 1 (head insert; SM‐A = no insert, SM‐B = head insert). In addition, (A) shows that while LC_17a/b_ isoforms are the same size (151 AAs), they differ in 5 of the last 9 COOH AAs. (B) The isoform‐specific differences of NMM. The NMMHC‐A and NMMHC‐B isoforms are similar in size (NMMHC‐A is 1,960 AAs long and NMMHC‐B is 1,976 AAs long). The NMMHC‐C isoform has an additional 20 AAs at the NH_2_ terminus (1,995 AAs). Sign ‘‐P‐’ means beginning non‐helical tail.

Indeed, pathological studies have found that the ratio of the stroma (composed mainly of SM) to the epithelium in normal human prostate is 2:1, while it is 5:1 in BPH patients 6. We also found the SM scattered throughout the rat prostate stroma and that contraction is significant generated in response to an α1‐adrenergic agonist 7. Generally, stimulation of the adrenergic nervous system 8 can increase intracellular Ca^2+^ concentration ([Ca^2+^]i) or the calcium sensitivity (when [Ca^2+^]i returned to basal levels), inducing thin filaments to slide past thick filaments (producing force) and preventing myosin dephosphorylation, which involves the RhoA/Rho‐kinase (ROK) mechanism (maintaining force) 9. Thin filaments are mainly composed of actin. Thick filaments are mainly composed of myosin, which is the motor molecule of the SM contractile apparatus. SM myosin (SMM) is composed of a pair of myosin heavy chains (MHCs) and two pairs of myosin light chains (LC_17_ and LC_20_) that are intimately intertwined 10. Both the 3′ and 5′ end of the MHC pre‐mRNA are alternatively spliced to generate COOH‐terminal isoforms (SM1 and SM2) and NH_2_‐terminal isoforms (SM‐A and SM‐B), respectively 11, 12. In addition, the essential light chain (LC_17_) is alternatively spliced and has two 3′ end isoforms (LC_17a_ and LC_17b_) 13, 14. As shown in Figure 1A, the isoform‐specific difference at the COOH terminus of the MHC is a length difference of 34 amino acids (AAs) (tail insert, SM1 is longer than SM2), while the isoform‐specific difference at the NH_2_ terminus of the MHC is 7 AAs at loop 1 (head insert, SM‐A = no insert, SM‐B = head insert). SMM with the head insert (SM‐B) and the tail insert (SM1) is 1,979 AAs long, whereas the SM MHC without the head insert (SM‐A) and the tail insert (SM2) is 1,938 AAs long. Meanwhile, LC_17a/b_ isoforms have the same size (151 AAs) but differ in five of the last nine COOH AAs. The SMM isoform composition has been demonstrated to affect force development 15 as well as force maintenance 16. The SM‐B, LC_17a_ and SM2 isoforms are associated with a faster more phasic‐type contraction (*e.g*. urinary bladder), whereas the SM‐A, LC_17b_ and SM1 isoforms are associated with a slower more tonic force generation (*e.g*. aorta) 17, 18, 19, 20, 21. We have demonstrated that the corpus cavernosum (CC) SM possesses a myosin isoform composition somewhat intermediate between bladder and aortic SM, considered to exhibit phasic and tonic characteristics, respectively 22. With regard to the prostate, no study has thoroughly characterized its SMM isoforms and correlating contractility profiles.

Similar to SMM, non‐muscle myosin (NMM) molecules are comprised of three pairs of peptides: two heavy chains, two regulatory light chains that regulate NMM activity and two essential light chains that stabilize the heavy chain structure. The NM MHC isoforms in mammalian cells result from three different genes (MYH9, MYH10 and MYH14) encoding NM MHC proteins (NMMHC‐A, NMMHC‐B and NMMHC‐C, respectively) 23, 24, 25, 26. As shown in a schematic figure (Fig. 1B), the NMMHC‐A and NMMHC‐B isoforms are more similar in size (NMMHC‐A is 1,960 AAs long and NMMHC‐B is 1,976 AAs long) to the SM MHC isoforms than NMMHC‐C (1,995 AAs). Indeed, the NMMHC‐C isoform has an additional 20 AAs at the NH_2_ terminus and shows significantly more variability in AA sequence than NMMHC‐A and NMMHC‐B 27. Although these myosin isoforms are referred as ‘non‐muscle’ myosin IIs to distinguish them from their muscle counterparts, they are also present in muscle cells, where they have distinct functions during skeletal muscle development and differentiation 28, as well as in the maintenance of tension of SM 29, 30. In addition, NMM is central in the control of cell migration and cytokinesis and tissue architecture owing to its position downstream of convergent signalling pathways 31, 32, 33, 34. A previous study found NMM expressed higher in embryonic or newborn tissues when compared to adult tissues 35, and the relative percentage of the NMM decreased significantly as the animals reached maturity 36. Thus, NMM might be known as embryonic myosin. Actually, NMM isoforms are expressed in a spatiotemporal manner during embryogenesis and adulthood. We also previously found that expression of NMMHC‐B was greatly increased by 4.5‐fold in bladder of partial bladder outlet obstruction rat model 37. Thus, NMM might be interacting with the pathophysiological process of some diseases. BPH has been suggested to be a ‘reawakening’ of embryonic processes in which embryonic prostatic mesenchyme dictates differentiation of new epithelial gland formation, which is normally seen only in foetal development 38, 39, 40, 41, 42. Therefore, NMM may indeed play important roles in BPH development.

At the functional level, blebbistatin (BLEB), a small cell permeable selective myosin II inhibitor, was originally discovered as the result of a high‐throughput screen for inhibitors of NMM 16. More recently, BLEB has been suggested to inhibit SM contraction with near equipotency as for NMM 43, 44, 45. Eddinger *et al*. 43 showed that force maintenance of chicken carotid artery was inhibited by BLEB (IC_50_, 3 μM). Also, our previous studies showed BLEB could inhibit the force maintenance of rat and human CC and bladder 44, 45. However, BLEB efficacy on prostate tension remains to be elucidated.

The aim of the current study was to determine SMM and NMM expression and isoform composition in the prostate, as well as their functional activities.

## Materials and methods

### Chemicals and tissues

All chemicals were purchased from Sigma‐Aldrich (St. Louis, MO, USA) except (±) BLEB and H‐1152 were from Tocris (Ellisville, MO, USA). The racemic mixture (±) of BLEB was used in all studies as it was determined that the active (−) enantiomer form was equipotent to the (±) racemic mixture in the *in vitro* studies and that the inactive (+) form did not induce significant relaxation 16, 43, 44, 46. A stock solution of (±) BLEB was made in dimethylsulphoxide (DMSO); the other substances were dissolved daily in double distilled water. Control experiments showed that the final concentration of 1/1000 (V/V) DMSO used in these studies did not significantly modify the relaxation response. Due to the known light sensitivity of BLEB, it was always kept in the dark in the refrigerator until just prior to usage and during the experiment, the organ bath chambers were kept covered. Male rat prostate, urinary bladder, CC and aorta were obtained from 10 Sprague Dawley rats weighing 300–350 g (Animal Center of Zhongnan Hospital of Wuhan University). All animal studies were approved by the research committee of Zhongnan Hospital of Wuhan University. Human prostatic smooth muscle cells (HPrSMCs) and epithelial cells (HPrECs) were purchased from Lonza (Walkersville, MD, USA). All strips including all three dimensions of approximately 1 cm were prepared for organ bath physiology studies and immediately placed in Krebs‐Henseleit (Krebs) solution with the rest of the tissue frozen in liquid nitrogen and saved at −80°C for subsequent molecular analyses or placed into 10% neutral buffered formalin for histological examination. All surgical procedures were performed under anaesthesia by intraperitoneal injection of sodium pentobarbital (35 mg/kg; Abbott Laboratory; Chicago, IL, USA).

### *In vitro* organ bath studies

As previously described 45, 47, rat prostate, bladder detrusor, CC and aorta strips were mounted longitudinally in a 4 ml organ bath—Multi‐Myograph Model 810MS (Danish Myo Technology; Aarhus, Denmark). The myograph was connected in line to a PowerLab 4/30 Data Acquisition System (ADInstruments; Colorado Springs, CO, USA) and in turn to a Dual‐Core processor Pentium computer for real‐time monitoring of physiological force. The SM strips were equilibrated at least 1 hr in Krebs buffer 45, 47 at 37°C with continuous bubbling of 95% O_2_ and 5% CO_2_. The buffer had the following mM composition: NaCl 110, KCl 4.8, CaCl_2_ 2.5, MgSO_4_ 1.2, KH_2_PO_4_ 1.2, NaHCO_3_ 25 and dextrose 11, and it was changed every 15 min. Strips were continuously adjusted to resting tension (0.5 g for rat prostate, 1.5 g for rat bladder, 0.35 g for rat CC and 0.7 g for rat aorta) 48, 49, 50, 51. After equilibration, the tissues were contracted with 60 mM KCl. This degree of contractile response was taken as 100% and the force induced by different concentrations of the various agonists (phenylephrine (PE) for prostate, CC, aorta and carbachol for bladder) was expressed as a percentage of this value. After washing several times to baseline with Krebs buffer, prostate strips pre‐contracted with 1 μM PE at a concentration pre‐determined to produce submaximal force were allowed to reach stable tension, and then, the relaxant effect of increasing doses of BLEB (1, 5, 10 μM), nitric oxide (NO) donor sodium nitroprusside (SNP) (10^−8^–10^−4^ M) and the Rho‐kinase inhibitor (H‐1152) (10^−9^–10^−5^ M) was evaluated.

### RNA extraction and cDNA synthesis

Total RNA was extracted using TRIzol reagent (Invitrogen, Carlsbad, CA, USA) according to the manufacturer's protocol. Briefly, the tissue was ground into a powder using a mortar and pestle cooled in liquid nitrogen, without allowing the tissue to thaw. The powder then was homogenized immediately in denaturing buffer using a T8 Ultra‐Turrax minielectric homogenizer (IKA Works; Wilmington, NC, USA), chloroform was added and mixed, the phases separated by centrifugation, and the RNA precipitated by isopropanol and then washed with 75% ethanol and dissolved in RNase‐free sterile water. The resulting RNA was quantitated by spectrophotometry at 260/280 nm. Total RNA (1 μg) then was reverse transcribed using 0.5 μg oligo (dT)_12–18_ primer, 500 μM dNTPs (Invitrogen) and 200 U of SuperScript II RNase H reverse transcriptase in a total volume of 20 μl for 50 min. at 42°C.

### Competitive reverse transcriptase polymerase chain reaction (competitive RT‐PCR)

As previously reported 20, 37, differences of nucleic acid sequence in each pair of SMM isoforms were quite small, so competitive RT‐PCR was applied to detect the expression of SMM isoforms. Polymerase chain reaction (PCR) was performed on 100 ng of the reverse transcribed cDNA using 2 units of Red Taq DNA polymerase (Sigma‐Aldrich), 200 ng each of upstream and downstream primer and 200 μM dNTPs (Invitrogen). SM‐A/SM‐B, SM1/SM2 and LC_17a_/LC_17b_ alternatively splice isoforms were amplified with competitive PCR, using a GeneAmp 9700 thermal cycler (Applied Biosystems, Foster City, CA, USA). The primer sequences are shown in Table 1. The cycling conditions were an initial 5 min. at 94°C followed by 35 cycles (30 sec. at 94°C, 30 sec. at 55°C and 120 sec. at 72°C), ended by a final one‐time 7‐min. incubation at 72°C to ensure extension of all products. The fact that all three pairs of myosin isoforms are generated *via* alterative splicing allowed us the opportunity to perform competitive PCR for each primer pair by designing primers that flanked the insert region.

**Table 1:** Primer sequences used to amplify target genes by PCR

Target gene	Primer	Primer sequence
SM‐A/‐B	Forward	5′‐GGCCTCTTCTGCGTGGTGGTC‐3′
	Reverse	5′‐TTTGCCGAATCGTGAGGAGTTGTC‐3′
LC_17a/b_	Forward	5′‐GAGAGTGGCCAAGAACAA‐3′
	Reverse	5′‐CAGCCATTCAGCACCATGCG‐3′
SM1/2	Forward	5′‐GCTGGAAGAGGCCGAGGAGGAATC‐3′
	Reverse	5′‐GAACCATCTGTGTTTTCAATAA‐3′
SM MHC	Forward	5′‐TTTGCCATTGAGGCCTTAGG‐3′
	Reverse	5′‐GTTCACACGGCTGAGAATCCA‐3′
NM MHC (MYH10)	Forward	5′‐TGAGAAGCCGCCACACATC‐3′
	Reverse	5′‐CACCCGTGCAAAGAATCGA‐3′
RPL19	Forward	5′‐GCGTCCTCCGCTGTGGTA‐3′
	Reverse	5′‐CATTGGCGATTTCGTTGGT‐3′

MHC: myosin heavy chain; NM: non‐muscle; SM: smooth muscle; LC: light chain; RPL19: ribosomal protein L19.

The PCR products were then separated by electrophoresis on a 2.5% agarose gel and were visualized using GelStar staining and ultraviolet illumination. Band density was quantified by reflectance scanning of gel photographs obtained with a BioDoc‐It camera set‐up (UVP; Upland, CA, USA) using a Bio‐Rad (Hercules, CA, USA) GS‐700 imaging densitometer and subsequent analyses using the Bio‐Rad Molecular Analyst 1D program that enabled us to obtain quantitative relative SMM isoform expression data for all isoform pairs.

### Real‐Time reverse transcriptase polymerase chain reaction (Real‐Time RT‐PCR)

As previously reported 37, RT products were also amplified in a 96‐well plate in a 25 μl reaction volume with all samples run in triplicate, using the model 7300 Real‐Time Thermocycler (Applied Biosystems). The following experimental protocol was utilized: denaturation (95°C for 10 min. to activate the polymerase) followed by an amplification programme repeated for 40 cycles (95°C for 15 sec., then 60°C for 60 sec.) using a single fluorescence measurement. SM MHC‐ and NM MHC‐targeted genes were amplified using SYBR Green for amplicon detection. For relative quantification, the efficiency of amplification for each individual primer pair (sequences shown in Table 1) was determined using cDNA target and the 2^−ΔΔct^ method 52 in conjunction with the RQ Study Software version 1.2.3 (Applied Biosystems). Gene expression was normalized to expression of the RPL19 ribosomal housekeeping gene as an internal control.

### SDS‐PAGE and Western blotting analysis

As previously described 47, proteins were extracted from frozen tissue samples using the CelLyticTM NuCLEARTM Extraction kit (amsbio; Abingdon, UK) and 30 μg of each sample was electrophoresed on a 10% SDS‐polyacrylamide gel and transferred to nitrocellulose membrane (Amersham Pharmacia; Piscataway, NJ, USA) by semidry electroblotting for 1 hr. The membrane was blocked for 2 hr at room temperature with 5% non‐fat dried milk dissolved in phosphate‐buffered saline (PBS) solution. The membranes were incubated overnight at 4°C with primary SM MHC (1:200, Santa Cruz, mouse monoclonal to MYH11, sc‐6956), NMMHC‐A (1:1000, Abcam, rabbit polyclonal to non‐muscle Myosin IIA, ab75590), NMMHC‐B (1:2000, Abcam, rabbit polyclonal to non‐muscle Myosin IIB, ab204358) and NMMHC‐C antibody (1:200, Santa Cruz, goat polyclonal to MYH14, sc‐138037). After washing several times with PBS, the membranes were incubated with antimouse or anti‐rabbit or anti‐goat immunoglobulin G (IgG) linked with horseradish peroxidase at a 1:10,000 dilution (Thermo Scientific Fisher; Rockford, IL, USA) for 1 hr at room temperature. Detection of reaction antigen was performed with an enhanced chemiluminescence (ECL) kit (Thermo Scientific Fisher). A monoclonal mouse antibody against GAPDH (1:5000; Sigma‐Aldrich) was used as a control to ascertain equivalent loading.

### Immunohistochemistry

Tissues fixed in 10% neutral buffered formalin for 24–36 hrs were routinely processed for paraffin embedding. Samples for immunohistochemistry (IHC) were sectioned at 5 μm and deparaffinized in xylene followed by descending grades of alcohols (100%, 95%, 70%, 30%). Antigen retrieval was performed in 10 mM sodium citrate buffer at pH 6.0, heated to 96°C, for 30 min., followed by proteinase K treatment for 10 min. Endogenous peroxidase activity was quenched using 3% hydrogen peroxide in PBS for 15 min. Blocking was performed by incubating sections in 5% normal donkey serum with 2% BSA for 1 hr. The sections were stained by routine IHC methods, using horse radish peroxidase polymer conjugate (Invitrogen), to localize the antibody bound to antigen, with diaminobenzidine as the final chromogen. All immunostained sections were lightly counterstained with haematoxylin. The primary antibody to myosin heavy chain (SM MHC) (1:100) was incubated for 1 hr at room temperature. Primary species (mouse IgG) was substituted for the primary antibody to serve as a negative control. Slides were evaluated for immunostaining by light microscopy.

### Confocal microscopy

HPrSMCs and HPrECs were resuspended in nutrient medium containing 10% foetal calf serum and a high concentration (15 mM) of thymidine to prevent cell division. Glass coverslips were coated with collagen (a drop of 1 mg/ml rat tail collagen in 5% acetic acid; Sigma Chemicals) and kept in 60‐mm tissue culture dishes overnight under UV light to polymerize the collagen. These dishes were rinsed with balanced salt solution to remove the acid, and the cells were plated in the culture dishes and fed with nutrient medium containing 10% foetal calf serum and 15 mM thymidine and incubated overnight. The next day, cells were attached on the collagen substratum coated on the coverslip. These cultures were fixed in 5% buffered formalin, kept in the refrigerator for 4 hr and processed for confocal microscopy.

Fixed cells were washed three times with PBS for 2 min. each time and incubated in blocking solution (1% BSA in PBS) for 30 min. Cells and rat prostate sections were then incubated for 1 hr at room temperature with the previously employed antibodies against NMMHC‐A (1:200), NMMHC‐B (1:100) or NMMHC‐C (1:50). The slides were then washed three times with PBS and reacted with anti‐rabbit or anti‐goat IgG made in goat labelled with TRITC. The slides were then washed three times in PBS and reacted with antibody specific to the whole SM MHC (1:100) labelled with FITC‐antimouse. Slides were washed three times in PBS, stained with DAPI for 3 min. to label nuclei and mounted in Vectashield mounting medium (Vector Laboratories; Burlingame, CA, USA), and the fluorescence signal was detected using confocal laser scanning microscopy (Carl Zeiss LSM 510, Carl Zeiss; Jena, Germany). Negative controls were prepared by replacing the primary antibody with preimmune serum.

### Statistical analysis

Results are expressed as mean ± S.E.M. for *n* experiments. Statistical analysis used either the Student's *t*‐test with Excel software (two sample treatments compared) or ANOVA and Bonferroni post‐tests with GraphPad Prism 5.0 (multiple means compared). *P *<* *0.05 was considered significant.

## Results

**Figure 2:**
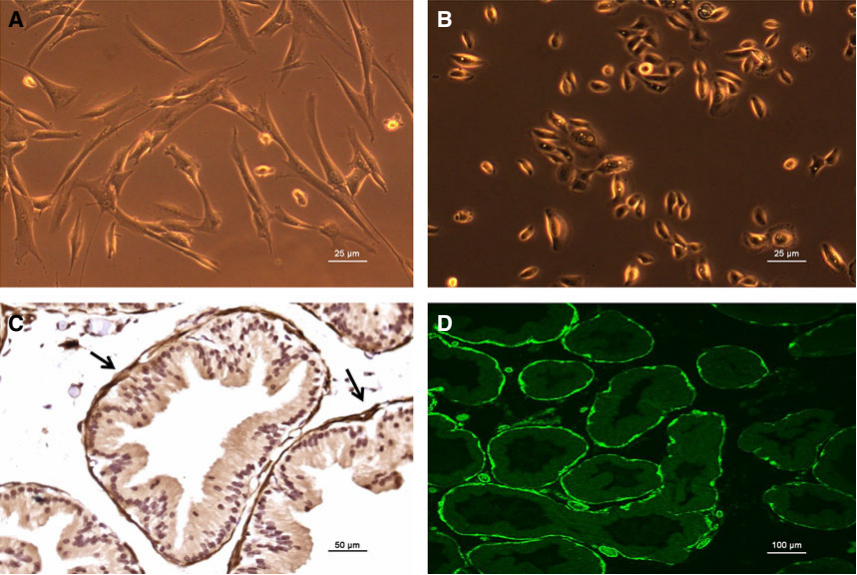
Morphology of human prostatic smooth muscle cells (HPrSMCs) and epithelial cells (HPrECs) and immunolocalization of smooth muscle (SM) myosin heavy chain (MHC) in ventral prostate. (**A**) and (**B**) display morphology under the light microscope of HPrSMCs and HPrECs, respectively. (**C**) and (**D**) display immunohistology and immunofluorescence of SM MHC in the rat prostate, respectively. The black arrows show SM in the outer stroma layer of rat prostate.

**Figure 3:**
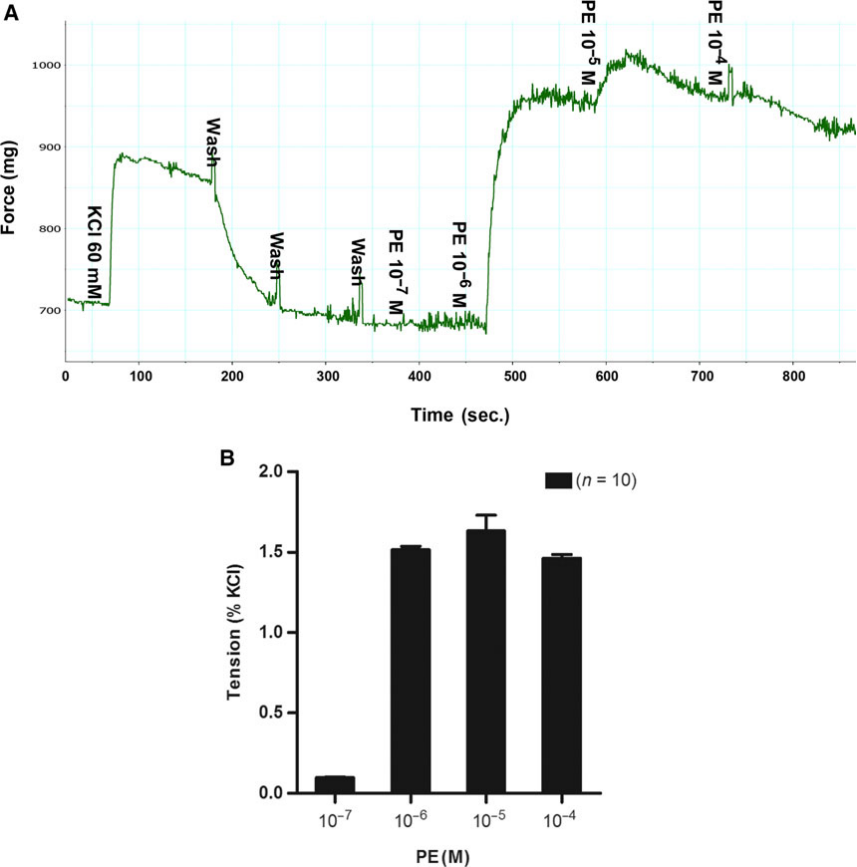
(**A**) The tracing of KCl induced contraction followed by phenylephrine (PE) induced dose–response contraction for rat ventral prostate. The *x*‐axis represents time (sec.), while the *y*‐axis represents force (mg). (**B**) Summary graph for *in vitro* contractility of rat ventral prostate smooth muscle (SM). The maximum response to 60 mM KCl was taken as 100%, while the contractile effect of cumulative concentrations (0.1–100 μM) of PE was evaluated as a percentage of this response. Values are expressed as the mean ± S.E.M. (*n *= strips obtained from 10 different animals).

**Figure 4:**
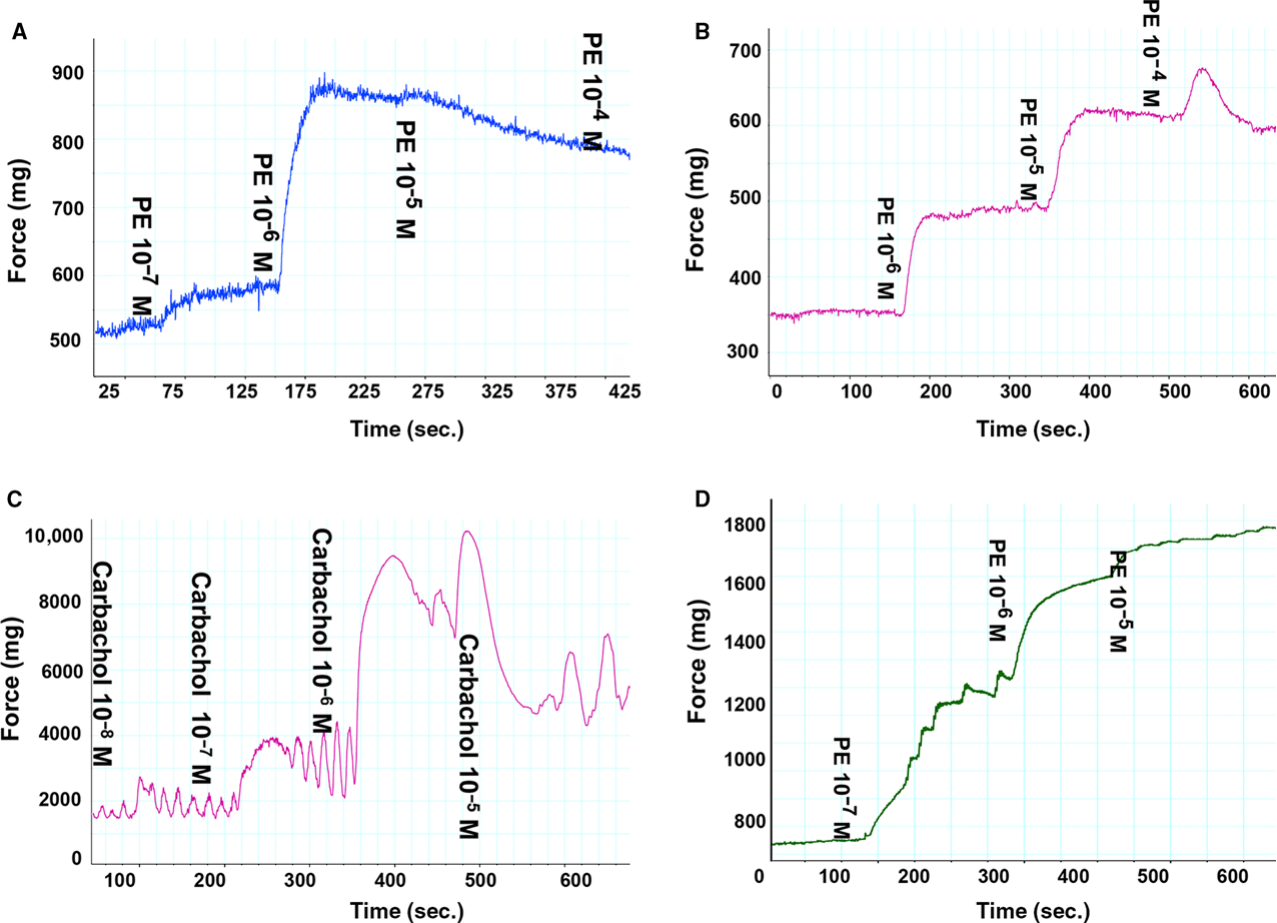
*In vitro* contractility of rat ventral prostate, corpus cavernosum (CC), bladder and aorta. (**A**–**D**) Curves of phenylephrine [for prostate (**A**), CC (**B**)] and aorta (**C**)) or carbachol [for bladder (**D**)] induced dose–response contraction. The *x*‐axis represents time (s), while the *y*‐axis represents force (mg).

The prostate is mainly composed of stroma (SM cell) and epithelium. Figure 2A and B are cultured human prostate SM and epithelium cells, respectively. As shown in Figure 2C and D, immunohistology and immunofluorescence demonstrated that prostatic SMM immunolocalized with the SM‐specific marker MHC was abundantly displayed in the rat prostate, predominantly in the outer stromal layer (black arrows). Thus, we investigated the contractility of prostate *in vitro*. Prostatic SM per se generated significant force in response to KCl depolarization and PE‐mediated adrenergic stimulation in a dose‐dependent manner. Figure 3A is representative tracing of rat prostate SM contraction in response to KCl and PE. The PE dose–response contractions were normalized to KCl‐elicited force and were averaged in Figure 3B. Isolated prostatic strips from rats produced 17 mg force/mg tissue tension at 60 mM KCl and reached maximal contraction at the concentration of 10^−5^ M of PE. At 1 μM, the prostate strip almost reached maximum contraction and this submaximal contraction was chosen for later experiments. Figure 4A showed rat prostate exhibits intermediate tonic and phasic contractile characteristics. This tonicity was further compared with rat CC (Fig. 4B), urinary bladder (Fig. 4C) and aorta (Fig. 4D). Consistent with previous reports, bladder and aorta produced typical phasic and tonic contractility, respectively. Similar to CC, prostate SM showed tonicity between bladder and aorta.

**Figure 5:**
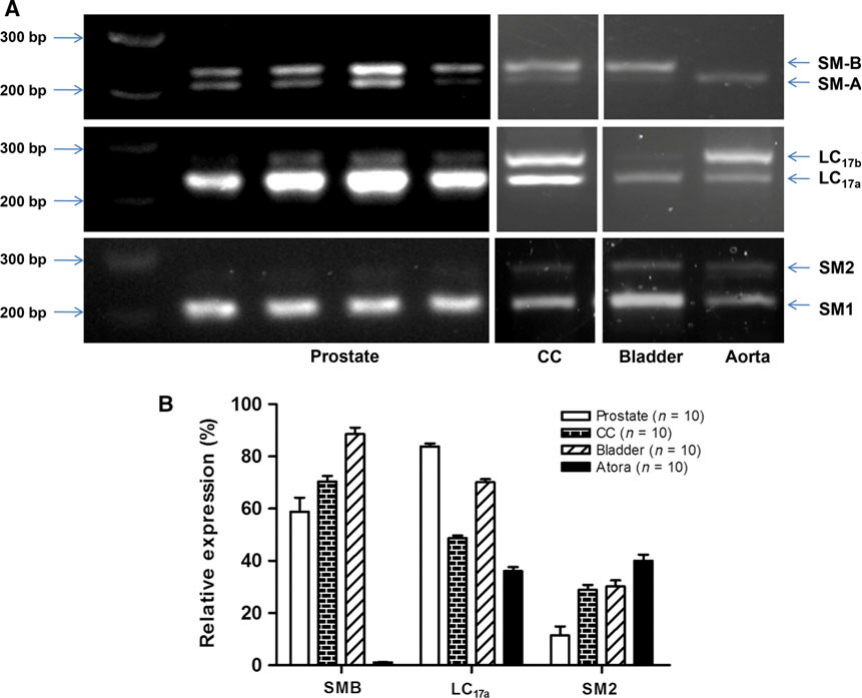
(**A**) RT‐PCR bands of smooth muscle myosin (SMM) isoforms in rat ventral prostate, corpus cavernosum (CC), bladder and aorta. Representative GelStar‐stained agarose gels of cDNA products resulting from competitive RT‐PCR analysis were utilized to detect the expression of SM‐A/SM‐B, LC_17a_/LC_17b_ and SM1/SM2 isoforms. Four lanes in the panel ‘prostate’ represent four different prostate samples. (**B**) The summary graph for the expression of SMM isoforms in rat ventral prostate, CC, bladder and aorta. Averaged quantitative determination of SMM pre‐mRNA mean isoform percentages in ventral prostate, CC, bladder and aorta was determined by reflectance scanning of gel photographs, and subsequent analyses were conducted by the Bio‐Rad Molecular Analyst 1D program. Values are expressed as mean ± S.E.M. (*n *=* *10 different animals for each group).

Subsequently, the compositions of SMM isoforms in the prostate, CC, bladder and aorta were examined with competitive RT‐PCR. In the current study, the SM‐A/B isoforms appeared to have a much wider range of expression levels than SM1/2 and LC_17a/b_ isoforms. As the relative ratios of SM‐B to SM‐A, LC_17a_ to LC_17b_ and SM2 to SM1 were associated with contraction characteristics, we focused upon determining the percentages of SM‐B, LC_17a_, SM2 in different tissues and found they were similar to each other in the same type of samples. As demonstrated in Figure 5A and B, rat aorta expressed exclusively SM‐A, less LC_17a_ and less SM2 favouring a slow tonic contraction (Fig. 4D). And rat bladder expressed 88.5% SM‐B, 70.1% LC_17a_ and 30.2% SM2 exhibiting a fast phasic contraction (Fig. 4C). Rat prostate contained almost similar SM‐B (58.8%), more LC_17a_ (83.8%) and less SM2 (11.4%) compared to their alternatively spliced counterparts. Similar to our previous report, rat CC contained more SM‐B (70.4%), almost equal LC_17a_ (48.7%) and less SM2 (28.9%). Interestingly, both prostate and CC SMM isoform compositions favoured an intermediate tonicity profile. Also correlated with SMM isoforms, time to 50% PE‐mediated maximum contraction for bladder, CC, prostate and aorta was 5.7 ± 1.2 S, 12.7 ± 2.5 S, 13.2 ± 2.6 S and 24.2 ± 2.4 S, respectively (Table 2).

**Table 2:** Time to 50% phenylephrine (PE) or carbachol‐mediated maximum contraction for prostate, corpus cavernosum (CC), bladder and aorta

Tissue	Number	Time (sec.)	*P* value (compared to Prostate)
Prostate	10	13.2 ± 2.6	NS
CC	8	12.7 ± 2.5	NS
Bladder	10	5.2 ± 1.2	<0.01
Aorta	7	24.2 ± 2.4	<0.01

CC: corpus cavernosum; NS: no significance; PE: phenylephrine.

**Figure 6:**
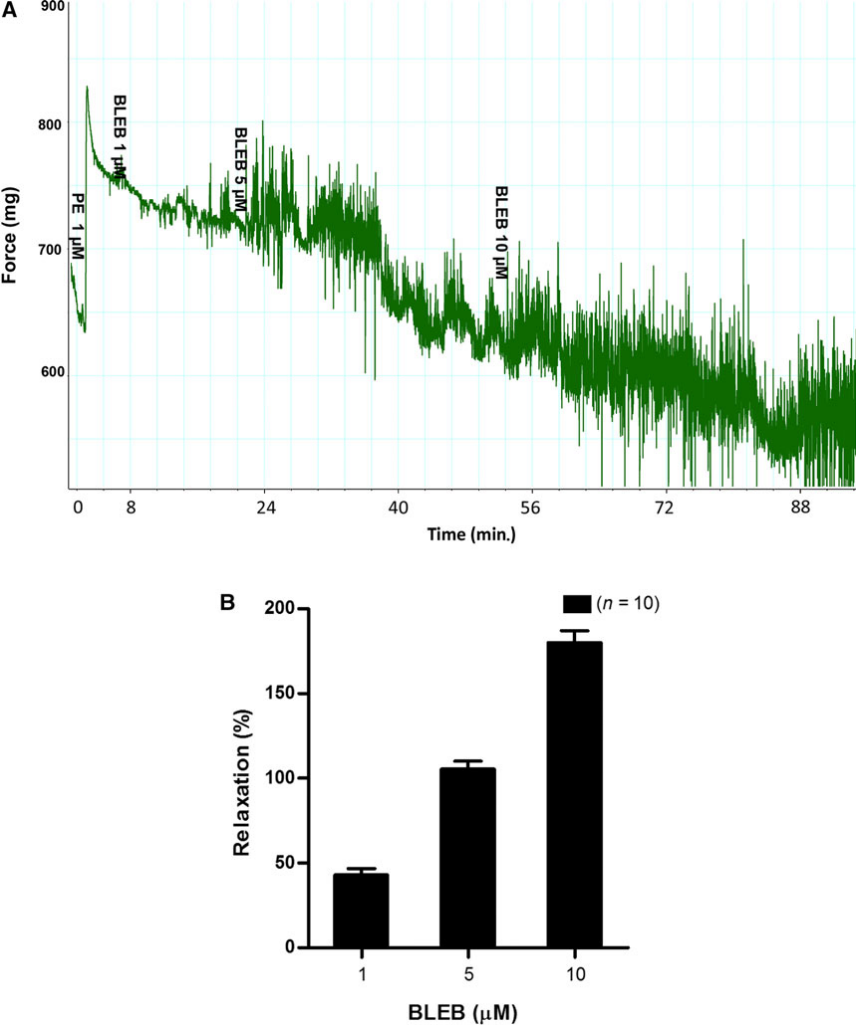
(**A**) The representative tracings of BLEB‐induced relaxation effects on ventral prostate pre‐contracted with PE. Ventral prostate strips pre‐contracted with 1 μM PE were allowed to reach stable tension, and then, the relaxant effect of increasing doses of BLEB (1, 5, 10 μM) was evaluated. The *x*‐axis represents time (min.), while the *y*‐axis represents force (mg). (**B**) Summary graph of BLEB‐induced relaxation effects on ventral prostate. Response to stimulus was taken as 100%, while the relaxation or inhibitory effect of BLEB was evaluated as a percentage of this response. Values are expressed as mean ± S.E.M. (*n *= strips obtained from 10 different animals).

**Figure 7:**
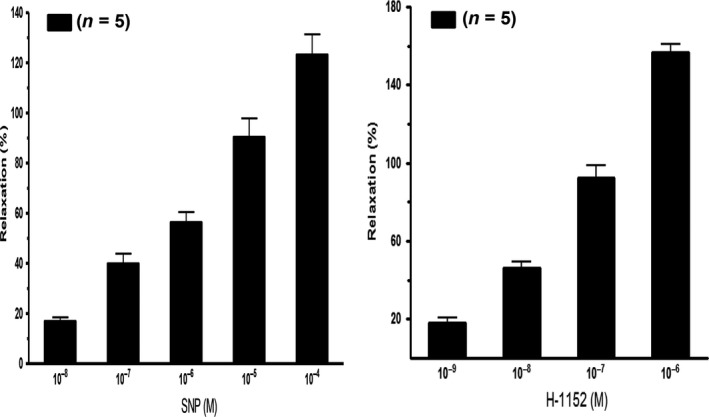
Summary graphs of nitric oxide (NO) donor sodium nitroprusside (SNP) and Rho‐kinase inhibitor H‐1152 on rat prostate pre‐contracted with phenylephrine (PE). The left panel and right panel display summary graphs of SNP and H‐1152 relaxation effects on PE‐pre‐contracted rat ventral prostate strips, respectively. Response to stimulus was taken as 100%, while the relaxation effects of SNP and H‐1152 were evaluated as a percentage of this response. Values are expressed as mean ± S.E.M. (*n *= strips obtained from five different animals).

We further investigated the efficacy of BLEB, a selective myosin II inhibitor, to relax rat prostate SM. As demonstrated in Figure 6A and B, BLEB strongly and dose‐dependently relaxed rat prostate strips. At 3 μM, BLEB almost completely attenuated the PE pre‐contracted prostatic strips. BLEB actually was capable of decreasing the tension lower than the baseline suggesting that BLEB could be useful for lowering basal prostate tone. In addition, both SNP and H‐1152 also strongly and dose‐dependently relaxed PE pre‐contracted rat prostate (Fig. 7).

**Figure 8:**
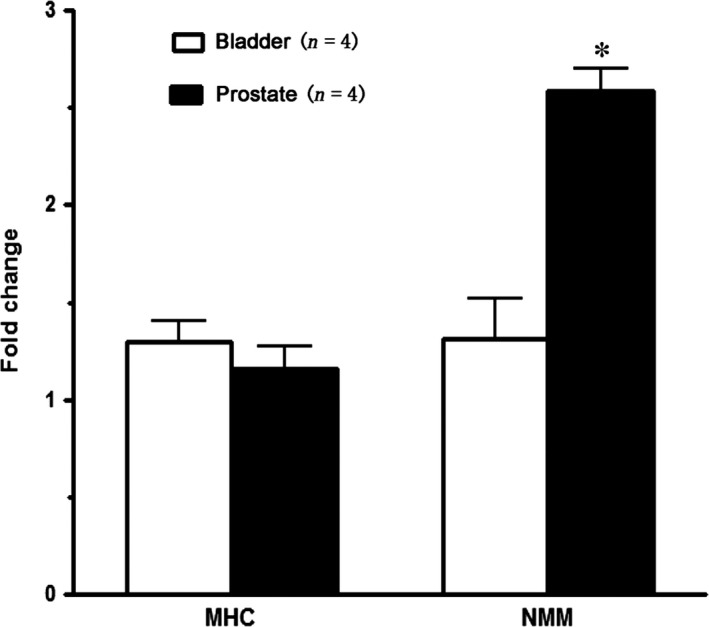
Expression of total smooth muscle myosin heavy chain (SM MHC) and non‐muscle myosin heavy chain B (NMMHC‐B) in rat ventral prostate and bladder. Total SM MHC and NMMHC‐B were quantified by real‐time RT‐PCR. Expression is normalized to the RPL19 housekeeping gene. Values are expressed as mean ± S.E.M. * equals *P *<* *0.05 (*n *=* *4 different animals for each group).

Real‐Time PCR was performed to quantify the relative expression of SMM (containing SM1/2 and SM‐A/B) and NMMHC‐B in rat prostate and bladder. As demonstrated in Figure 8, NMMHC‐B expression in rat prostate was more than 2‐fold higher than that of rat bladder, while SMM expression was found to be similar.

**Figure 9:**
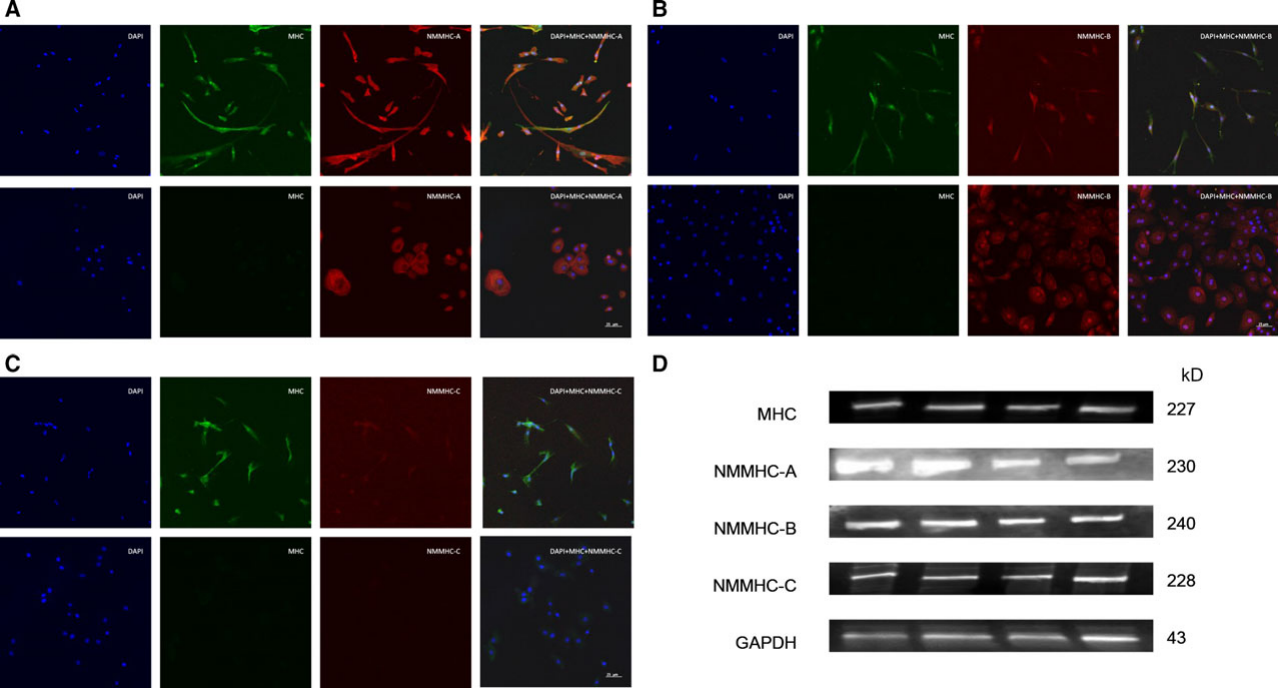
Immunolocalization of SM MHC and NM MHC isoforms in HPrSMCs and HPrECs and expression of SM MHC and NM MHC isoforms in rat prostate. HPrSMCs (upper subpanels of **A**,** B** and **C**) and HPrECs (lower subpanels of **A**,** B** and **C**) were processed as described in the Materials and methods. Double immunofluorescence staining for NMM isoforms (NMMHC‐A, NMMHC‐B and NMMHC‐C) (red fluorescence) and SM MHC (green fluorescence) was conducted in HPrSMCs and HPrECs. Nuclei were stained by DAPI (blue). The images were photographed by confocal fluorescence microscopy. The scale bars for (**A**)–(**C**) are 25 μm. (**D**) Representative Western blotting bands (from top to bottom) of SM MHC and NMM isoforms (NMMHC‐A, NMMHC‐B and NMMHC‐C) and GAPDH in rat ventral prostate.

**Figure 10:**
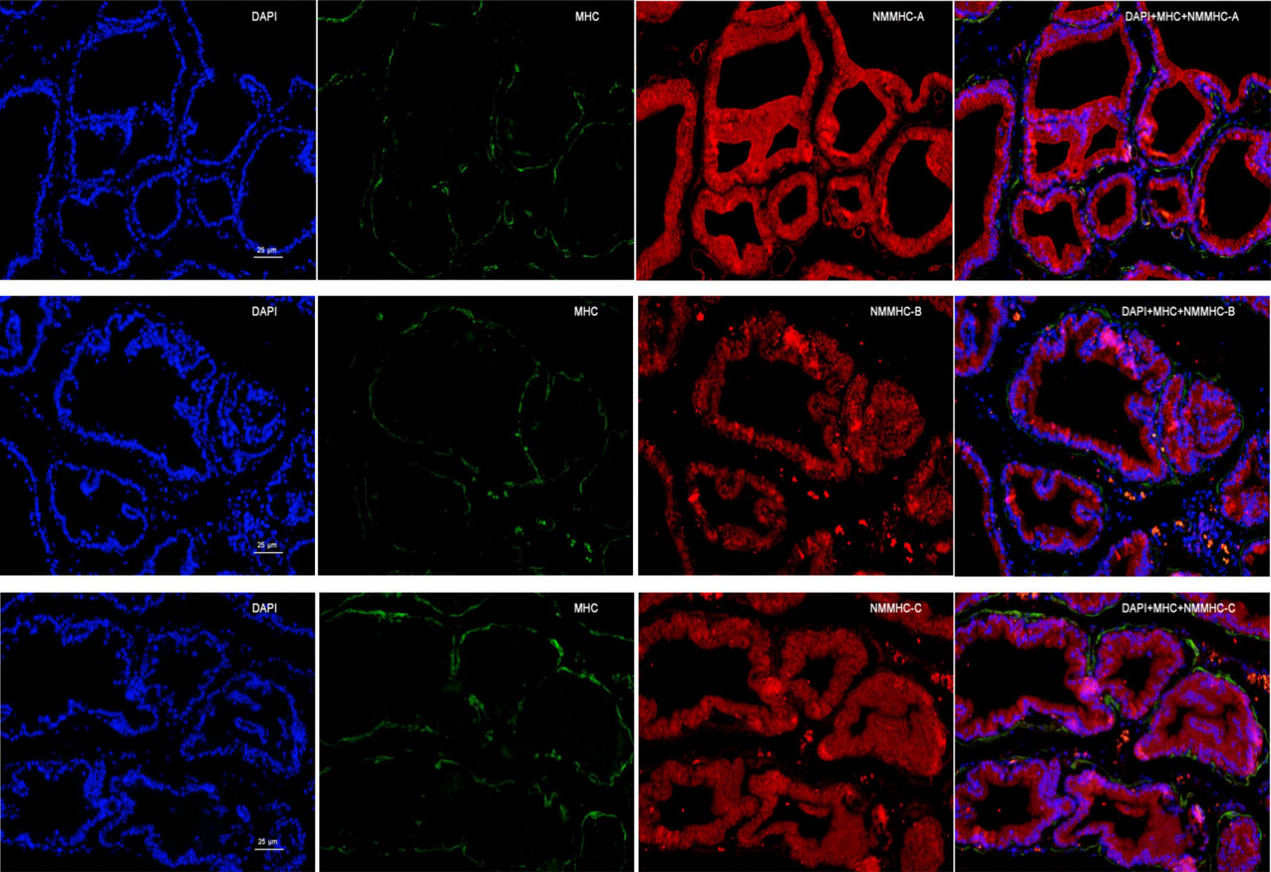
Immunolocalization of SM MHC and NM MHC isoforms in rat prostate. Representative double immunofluorescence staining for NMMHC‐A (top), NMMHC‐B (middle) and NMMHC‐C (bottom) (red fluorescence) and SM MHC (green fluorescence) was conducted in rat prostate. Nuclei were stained by DAPI (blue). The images were photographed by confocal fluorescence microscopy. The scale bars represent 25 μm.

In addition to SMM, NMM isoforms were also determined in the prostate. We used cultured HPrSMCs and HPrECs to accurately localize the SM MHC and NM MHC expression in human prostate cells. Immunofluorescence showed SMM was abundantly present only in HPrSMCs (Fig. 9A–C). NMMHC‐A and NMMHC‐B were both present in HPrSMCs and HPrECs (Fig. 9A and B), while NMMHC‐C was found only in HPrSMCs and no expression in HPrECs (Fig. 9C). As demonstrated in Figure 9D, all NMM isoforms (NMMHC‐A, B, C) were abundantly detected in rat prostate with Western blotting analysis. To accurately localize the SM MHC and NM MHC expression in rat prostate, we did immunofluorescence studies of rat prostate and found that all NMM isoforms (NMMHC‐A, B, C) were abundantly detected in the stroma and epithelium of rat prostate and SM MHC was located in the stromal layer (SM cells) (Fig. 10).

## Discussion

Our novel data demonstrated the expression of isoforms of SMM and NMM and their functional activities in the prostate. Rat prostate mainly expressed LC_17a_ and SM1 but with relatively equal expression of SM‐B at the mRNA level, correlating with an intermediate tonicity between phasic bladder and tonic aorta with a rapid increase in force development but also with extended force maintenance at the functional level. Meanwhile, isoforms of NMMHC‐A, B, C were also abundantly detected in rat prostate with SMM present only in SM, NMMHC‐A, B, C isoforms both present in stromal and endothelial cells in rat prostate. But in human prostatic cell lines, NMMHC‐C expressed only in the SM cells, which was different to rat prostate. At the functional level, the SMM selective inhibitor BLEB could potently relax rat prostate SM, comparable to SNP and H‐1152.

The prostatic SM represents a significant component of the stroma. In the present study, it was identified using the highly specific SM marker MHC and its spatial arrangement in the stroma was uncovered. It is assumed this arrangement is not optimal for force generation. However, we observed that it did produce significant force in response to either KCl or PE (Figs 3A and 4A). Thus, the active forces in the prostatic tissue might play an important role in the pathophysiology of BPH. However, until now the contractile properties of prostatic SM have not been well characterized. Lin *et al*. 53 characterized the expression of SM MHC and NM MHC at both peptide and mRNA levels in human prostate tissue and only demonstrated that both SM1 and SM2 isoforms are expressed in prostate but not in fibroblast cells. In current study, we thoroughly characterized rat prostate SM isoforms and found it expressed almost equal SM‐B (58.8%), mainly LC_17a_ (83.8%) and less SM2 (11.4%). When compared to rat CC SM which expressed SM‐B (70.4%), almost equal LC_17a_ (48.7%) and less SM2 (28.9%), a difference was demonstrated between prostate and CC. Thus, there may be organ level differences in SM myosin isoform composition among these different tissues. However, at the functional level, both prostate and CC SM displayed an intermediate contraction phenotype between phasic bladder and tonic aorta (Fig. 4).

Previous studies demonstrated the SM‐B isoform was associated with SM tissues with a more phasic contractile nature, faster shortening velocity and higher ATPase activity, whereas the SM‐A isoform was found in a slower and more tonic type SM with lower ATPase activity 17, 18, 19, 20. Similarly, the relative higher ratio of the LC_17a_ to LC_17b_ isoform was also demonstrated a more phasic contraction 18, 54, 55. Indeed, the present study showed that rat aorta expressed exclusively SM‐A and less LC_17a_ favouring a slow tonic contraction (Fig. 4D), while rat bladder expressed almost exclusively SM‐B and predominantly LC_17a_ exhibiting a fast phasic contraction (Fig. 4C). Consistent with these observations, the time to 50% maximum contraction was 24.2 sec. for aorta, which was 4‐fold longer than that for bladder. Also consistent was the demonstration that the rat prostate expressed almost equal SM‐B relative to SM‐A correlating with an intermediate contraction phenotype and time to 50% maximum contraction between bladder and aorta (Table 2), although over 80% LC_17a_ was detected. It was reported the effect of LC_17_ isoforms in shortening velocity might be confounded by differences in SM‐A/B isoforms. Interestingly, Rovner *et al*. 56 reported an approximate 2‐fold higher *in vitro* motility speed and Mg^2+^‐ATPase activity in expressed SM‐B heavy meromyosin regardless of the LC_17_ isoforms present (pure LC_17a_ or pure LC_17b_). Further supporting the Rovner *et al*. observation, Sweeney *et al*. 57 reported that *in vitro* motility sliding velocity and actin‐ATPase activity correlated with increasing loop size/flexibility but were not affected by the LC_17_ isoforms present. X‐ray crystallography data have revealed that the LC_17_ subunits can approach the 25/50‐kD loop, which is the location of the NH2‐terminal SM‐A‐/B isoforms and near the nucleotide binding pocket of myosin and regulates the opening for the nucleotide binding cleft 54, 57. Although it is unclear whether this is a physiologically relevant observation, it is intriguing because a possible interaction between the variable sizes of SM‐A/B isoforms and LC_17a/b_ isoforms could help resolve the apparent discrepancy in the data for a possible role of the LC_17_ isoforms in the regulation of unloaded shortening velocity in SM tissues. The SM MHC tail isoforms (SM1 and SM2) in rat prostate, CC, bladder and aorta showed differences in levels of expression, but there seemed to be no obvious correlation with muscle shortening velocity in our study. Sparrow *et al*. 58 also concluded that there are no obvious differences in the intrinsic properties of these two isoforms. They found a small positive correlation between SM1 expression and unloaded shortening in skinned rat myometrial muscle, but a small inverse correlation was confirmed in intact tissue. This discrepancy might be due to inherent differences in these two model systems.

In contrast to the role of SMM in mediating contraction, NMM had been proposed to be involved in cellular ‘housekeeping’‐type processes, including proliferative, synthetic and secretory functions 27, 59, 60. In the present study, we found that NMMHC‐A, B and C were present both in SM and endothelial cells of rat prostate, and NMMHC‐A and NMMHC‐B were present in HPrSMCs as well as HprECs, However, NMMHC‐C was expressed only in the HPrSMCs. Golomb *et al*. also found that NMMHC‐C is expressed in the apical area of epithelial cells in the inner ear of mouse. Thus the different location of NMMHC‐C in SM or endothelial cells might attributable to the different tissue from different species. As NMMHC‐A had the highest rate of ATP hydrolysis of the three NMM isoforms 28 and NMMHC‐B played an ancillary role in SM contraction, they might have distinct functions in the maintenance of tension during tonic contractions 29, 30. In our current study, we found NMMHC‐B expressed more than 2‐fold higher in rat prostate than in rat bladder. It was further suggested that NMM might also play an important role in cell proliferation and BPH development. Interestingly, NMMHC‐C is the newest identified NMM isoform with presently unknown roles. Therefore, it seems to be clearly worth determining the functional activity of these NMM isoforms in the prostate in the future.

Finally, we showed PE pre‐contracted prostate SM can be potently relaxed by BLEB (a selective myosin inhibitor), comparable to the effect of SNP (NO donor) and H‐1152 (a specific, strong and membrane‐permeable inhibitor of Rho‐kinase). Although BLEB was originally reported to be a selective inhibitor of the myosin II isoforms expressed by striated muscles and non‐muscle (IC_50_ = 0.5–5 μM) but a poor inhibitor of purified turkey smooth muscle myosin II (IC_50_ ∼80 μM) 46, our previous studies have clearly demonstrated that BLEB is indeed also a potent inhibitor for rat bladder and CC SM 44, 45. In current study, we found that BLEB is a potent inhibitor for rat prostate SM and can significantly relax prostate SM at a concentration of 3 μM, comparable with the range of IC_50_ values (∼3–5 μM) for SM of chicken arteries reported by Eddinger *et al*. Interesting to note is that Eddinger *et al*. 43 also found that contraction of chicken gizzard was less potently inhibited by BLEB. Thus, an additional protein was assumed to uniquely exist in avian gizzard and reduced the effectiveness of BLEB. As it has been suggested that BLEB efficacy may be impacted by the relative expression of the SM‐A/B isoforms composition 37, the more relatively expressed SM‐B in the prostate could contribute to the potent relaxing effect of BLEB for this kind of SM. In addition, BLEB was originally discovered as a selective inhibitor of NMM 16 and the abundant expression of NMMHC‐A and B isoforms in the prostate SM found in current study could also contribute to the strong inhibitory ability of BLEB in this tissue. As NMM plays an important role in cell proliferation, it is plausible that inhibiting or knocking down NMM could prevent prostate growth. Indeed, we found atrophy of prostate when BLEB was injected into rat prostate *in vivo* (data not shown). Therefore, prostate SMM or NMM could be a promising target for discovering new drugs for treating BPH.

A limitation for the current study is that the protein levels of SMM isoforms were not determined as isoform‐specific antibodies are not commercial available at present. However, a previous study demonstrated that mRNA levels of SMM isoforms correlated well with protein expression 61. Another weakness is that the functional activities of SMM and NMM isoforms cannot be distinguished in the prostate, although their isoforms composition was accurately determine.

In conclusion, we demonstrated, for the first time, the expression, characterization, composition and functional activities of SMM and NMM isoforms in the rat prostate. It is suggested that the isoforms of SMM and NMM could play important roles in the development and clinical effects of BPH.

## Funding source

X.H.Z. is supported by National Natural Science Foundation of China (Nos. 81270843 and 81160086).

## Conflict of interest

The authors declare that they have no competing interests.
